# Trend of carbapenemase changes and epidemiological distribution of carbapenem-resistant *Klebsiella pneumoniae* clinically isolated in a hospital: a single-center retrospective study from 2015 to 2024

**DOI:** 10.3389/fpubh.2026.1944879

**Published:** 2026-10-21

**Authors:** Zhi Wang, Huimin Hu, Shuyun Wang, Donghong Yin, Jinju Duan, Jianbang Kang

**Affiliations:** 1 School of Pharmacy, Shanxi Medical University, Taiyuan, China; 2 Medicinal Basic Research Innovation Center of Chronic Kidney Disease, Ministry of Education, Shanxi Medical University, Taiyuan, China; 3 Department of Pharmacy, Second Hospital of Shanxi Medical University, Taiyuan, China

**Keywords:** carbapenemase epidemiology, carbapenem-resistant *Klebsiella pneumoniae*, epidemiological distribution, public hospitals, retrospective study

## Abstract

**Background:**

The spread of carbapenem-resistant *Klebsiella pneumoniae* (CRKP) has become a concerning threat. In particular, infections caused by CRKP are on the rise globally, posing a major public health challenge to human health. In China, KPC and NDM are the main types of carbapenemases among CRKP strains.

**Methods:**

We collected 324 strains of CRKP clinically isolated from a Grade A tertiary hospital between 2015 and 2024. Antibiotic susceptibility was tested using the Antibiotic susceptibility was tested using the Kirby-Bauer disk diffusion method and agar dilution method. The carbapenemase phenotypes were confirmed by the modified carbapenem inactivation method (mCIM) and the EDTA-modified carbapenem inactivation method (eCIM). And we detected carbapenemase-encoding genes were detected by polymerase chain reaction (PCR). Analyzed the changing trends of carbapenemases in CRKP strains. Besides, we divide CRKP patients into four groups. Then we analyze the distribution differences in the length of hospital stay, total drug cost, antibacterial drug cost, and outcomes among patients in each group.

**Results:**

Within the decade from 2015 to 2024, there were significant changes in the carbapenemase types of CRKP. Since 2021, the predominant carbapenemase type in the strains has shifted from NDM to KPC. Among all the included CRKP strains, isolates recovered from pulmonary infection sites and sputum specimens accounted for the largest proportion, totaling 206 strains. The intensive care unit (ICU) yielded the highest number of CRKP strains, with a total of 78 isolates. Intra-group analysis showed that there were statistically significant differences in the length of hospital stay of CRKP patients in the two dimensions of specimen classification and disease classification (*χ*^2^_disease_ = 20.899, *p* < 0.001; *χ*^2^_specimen_ = 23.586, *p* < 0.001). There were statistically significant differences in the total drug cost in the three dimensions of disease classification, department classification, and severity classification (*χ*^2^_disease_ = 21.224, *p* < 0.001; *χ*^2^_department_ = 45.711, *p* < 0.001; *χ*^2^_severity_ = 9.571, *p* = 0.023). There were statistically significant differences in the antibacterial drug cost in the three dimensions of disease classification, severity classification, and specimen classification (*χ*^2^_disease_ = 22.916, *p* < 0.001; *χ*^2^_department_ = 20.711, *p* < 0.001; *χ*^2^_specimen_ = 14.811, *p* = 0.022). There was a statistically significant difference in the treatment outcomes in the one dimension of severity classification (*χ*^2^_severity_ = 14.729, *p* < 0.001).

**Conclusion:**

In hospital, the type of carbapenemase produced by CRKP strains has gradually shifted from being predominantly NDM to predominantly KPC over the past decade. The disease types, inpatient departments, severe illness conditions, and sampling specimens of CRKP patients have all had impacts on the patients treatment outcomes.

## 1 Introduction

*Klebsiella pneumoniae*, which ranks among the most prevalent multidrug-resistant pathogens in clinical settings, is commonly associated with pneumonia, bloodstream infections, urinary tract infections, and surgical site infections, especially in critically ill patients (1). Carbapenem-resistant *Klebsiella pneumoniae* (CRKP) has emerged as one of the most critical public health threats worldwide in recent years, for which antibiotic options remain limited (2).

The primary resistance mechanism of CRKP involves the production of carbapenemases, which predominantly include the *Klebsiella pneumoniae* carbapenemase (KPC), the New Delhi metallo-β-lactamase (NDM), and oxacillinase-48 (OXA-48) (3). Carbapenem antibiotics serve as the last line of defense against multidrug-resistant bacteria, and these enzymes play a critical role in mediating *Klebsiella pneumoniae* resistance to such agents (4). In China, NDM and KPC represent the two primary carbapenemases responsible for CRKP resistance, yet their geographical distribution exhibits considerable heterogeneity (5). For instance, among 50 resistant isolates from Fujian Province, 48 strains (96%) carried KPC. In contrast, NDM was identified in 74 out of 76 carbapenem-resistant isolates (97%) obtained from Shaanxi Province (6). The significant regional variations in carbapenemase distribution underscore the necessity of conducting region-specific studies on CRKP enzyme production.

According to the report issued by the China Antimicrobial Resistance Surveillance System, the incidence of CRKP in China has remained largely stable in recent years, showing a slight decline from 10.9% in 2020 to 10% in 2024 (7).^1^ This represents an improvement compared with the upward trend from 5.6% in 2014 to 10.9% in 2020, which was documented in relevant studies at the time (8). Meanwhile, in a ten-year retrospective analysis, the number of CRKP strains in our hospital also showed a significant upward trend. These findings indicate that the dissemination of CRKP strains continues to demand close attention (9). Therapeutic strategies for CRKP infections are significantly influenced by the type of carbapenemase produced (10). Ceftazidime-avibactam (CZA) is considered an optimal choice for treating CRKP strains harboring KPC enzymes (11). This β-lactam/β-lactamase inhibitor combination exerts inhibitory activity against class A, class C, and certain class D β-lactamases (including OXA-10 and OXA-48-like carbapenemases), but remains ineffective against class B metallo-β-lactamases (MBLs) such as NDM and IMP (12). The treatment of NDM-producing isolates typically requires combination therapy with aztreonam, which maintains stability against metallo-β-lactamases (13). Currently, although there are national surveillance data in China showing the overall situation of CRKP infections, there is still a lack of in-depth reports on regional and long-term continuous molecular epidemiological surveillance data, especially the dynamic change rules of the proportion of carbapenemase types. Systematically revealing the long-term change trends of carbapenemase types of CRKP in specific medical institutions is crucial for guiding local infection prevention and control, optimizing empirical treatment, and the rational use of new antibacterial drugs. This study screened 324 CRKP isolates collected from a tertiary hospital in Shanxi Province, China between 2015 and 2024. Carbapenemase phenotypes were confirmed using the modified carbapenem inactivation method (mCIM) and the EDTA-modified carbapenem inactivation method (eCIM). The analysis aimed to characterize the shifting trends in carbapenemase production among CRKP strains within this hospital setting, thereby informing the development of effective infection prevention and control measures.

Current prognostic studies on CRKP patients have predominantly focused on variables such as patient age and laboratory parameters, while paying less attention to clinical factors including underlying diseases, hospital departments, specimen types, and critical illness status (14, 15). This study is a retrospective analysis. In addition to analyzing the changing trends of carbapenemases in CRKP strains from a certain hospital. Non-parametric tests between groups were conducted using the Kruskal-Wallis H test and the Bonferroni - corrected Mann - Whitney U test to identify the risk factors for treatment outcomes.

## 2 Materials and methods

### 2.1 Description of bacterial isolates

A retrospective analysis was conducted on 324 unique carbapenem-resistant *Klebsiella pneumoniae* (CRKP) isolates obtained between 2015 and 2024 from the Second Hospital of Shanxi Medical University, a large 3,200-bed tertiary hospital in Shanxi Province. CRKP is defined as *Klebsiella pneumoniae* that demonstrates resistance or intermediate resistance to at least one of the following antibiotics: meropenem or imipenem or ertapenem. Strain data were obtained from the original isolation records of the hospital LIS system. The study encompassed patient demographics, specimen types, and excluded duplicate isolates from the same infection site in individual patients. Strain characteristics were correlated with patient clinical information and summarized annually.

The patients were categorized using four classification approaches, namely based on severity type, disease type, department type and specimen type. Patients were divided into 3 groups based on severity type: patients with sepsis and septic shock, patients with severe pneumonia, and patients with other relatively mild infections. According to the primary infection site, cases were classified into 6 groups by disease type. In terms of department type, patients were categorized into 8 groups based on their admitting inpatient department. For specimen type, cases were assigned into 7 groups according to the clinical specimen from which the CRKP strain was isolated. The definitions of infection sites are as follows: Pulmonary infection was defined as the presence of new or progressive infiltrate, consolidation, or cavitation on chest imaging, accompanied by at least one infection-related clinical manifestation (16). Bloodstream infection was defined as the presence of recognized clinical signs of systemic infection with at least one positive blood culture growing a bacterial pathogen not related to an infection at another site (17). Urinary tract infection was defined by the presence of at least one local urinary tract symptom with no other identified non-infectious cause (18). Intra-abdominal infection was defined as the presence of clinical signs and imaging evidence of intra-abdominal infection (19). Central nervous system infection was defined as the presence of new-onset neurological dysfunction, meningeal irritation signs, or altered mental status, with consistent neuroimaging or cerebrospinal fluid inflammatory abnormalities. For cases with multi-site infection, classification and statistical analysis were performed according to the location of the primary infectious source. For patient treatment outcomes, we make the assessment at discharge: outcomes of cured and improved are defined as effective treatment, while outcomes of death and unhealed and worsened and non-medical discharge and abandonment of treatment etc. are defined as ineffective treatment.

The resistance levels of these CRKP isolates to various carbapenem antibiotics, including imipenem and meropenem. Was determined using the using the Kirby-Bauer disk diffusion method and the agar dilution method (20). All isolates were subjected to reidentification through MALDI-TOF MS. Additionally, the isolates were preserved at −80 °C to facilitate long-term storage and support subsequent antimicrobial susceptibility testing. During the study period, the strain identification methods in the clinical microbiology laboratory of our hospital have remained consistent throughout the study period, while the antimicrobial susceptibility testing methods were updated in accordance with the latest prevailing standards at the corresponding time points. The CRKP screening strategy was routine testing of clinically submitted specimens, with no significant change in screening intensity. The number of hospital beds was basically stable. There were no major adjustments to specimen submission specifications.

### 2.2 The inclusion criteria of bacterial isolates

We used a bacterial isolation database established by the Clinical Microbiology Laboratory of the Second Hospital of Shanxi Medical University. All patients with microbiological confirmation of *Klebsiella pneumoniae* between January 2015 and December 2024 were included in the study. The inclusion criteria are as follows: (1) only strains isolated from inpatients are included; (2) only the first isolate from the same patient are included. Following the results of carbapenemase phenotypic identification, we subsequently included *Klebsiella pneumoniae* strains that were definitively identified as CRKP strains. To distinguish between infection strains and colonization strains, we established a predefined rule that only cases where the CRKP strain isolation site matches the primary infectious focus of the patient can be included in the final study cohort. All strains meeting the inclusion criteria were included in the analysis, with no selective exclusion.

### 2.3 Phenotypic detection of carbapenemases and resistance mechanism

Carbapenemase production was evaluated using the modified carbapenem inactivation method (mCIM) and the EDTA-modified carbapenem inactivation method (eCIM), in accordance with the Clinical and Laboratory Standards Institute (CLSI) M100 S31 guidelines (20). Briefly, for each tested isolate, the mCIM procedure involved emulsifying a 1 μL loopful of an overnight blood agar culture in 2 mL of Tryptic Soy Broth (TSB) (Oxoid, Oxfordshire, UK). After vortexing for 10 s, a 10 μg meropenem disk (Oxoid, Oxfordshire, UK) was added to the broth, which was then incubated at 35 °C in ambient air for 4 h. Subsequently, a *Escherichia coli* ATCC 25922 suspension adjusted to a 0.5 McFarland standard was inoculated onto a Mueller-Hinton Agar (MHA) (Oxoid, Oxfordshire, UK) plate. The meropenem disk was retrieved from the TSB using a 10 μL loop and placed onto the MHA plate seeded with *E. coli* ATCC 25922. During this procedure, care was taken to minimize residual TSB liquid. The MHA plates were incubated inverted at 35 °C in ambient air for 18 to 24 h. For each isolate, an additional 2 mL TSB tube was prepared for the eCIM assay. Subsequently, 20 μL of 0.5 M EDTA was added to the 2 mL TSB to yield a final EDTA concentration of 5 mM, after which the steps of the mCIM procedure were repeated. Parallel evaluation of the mCIM and eCIM tubes was performed by placing the meropenem disks from both tubes onto a single MHA plate inoculated with the *E. coli* ATCC 25922 indicator strain. Control experiments used *K. pneumoniae* ATCC BAA 1705 as a positive control, which was expected to show carbapenemase activity, while *K. pneumoniae* ATCC BAA 1706 served as a negative control.

The polymerase chain reaction (PCR) was used to detect the genes encoding carbapenemases(bla-KPC, bla-NDM, bla-IMP, bla-VIM, and bla-OXA-48). In brief, for each tested isolate, genomic DNA was isolated and purified from clinical CRKP isolates using the Ezup Column Bacterial DNA Extraction Kit. Common carbapenemase-associated resistance genes were amplified by PCR. After PCR amplification, the products were separated and identified by agarose gel electrophoresis, and the positive bands were subjected to Sanger sequencing. The obtained sequence data were submitted to the NCBI online BLAST platform^2^ for homology alignment.

### 2.4 Antimicrobial susceptibility testing

Antimicrobial susceptibility testing was performed using the using the Kirby-Bauer disk diffusion method and the agar dilution method, which assessed the susceptibility of CRKP strains to 7 distinct antimicrobial agents: Imipenem, Meropenem, Cefoperazone-Sulbactam Amikacin, Aztreonam and Tigecycline. Minimum inhibitory concentrations (MICs) were determined following the Clinical and Laboratory Standards Institute (CLSI) 2023 guidelines. Besides, Colistin susceptibility testing was performed using the broth microdilution method, with results interpreted according to European Committee on Antimicrobial Susceptibility Testing (EUCAST version 10.0) criteria. For tigecycline, the breakpoint against Enterobacterales was defined per United States Food and Drug Administration (FDA) standards, where isolates with an MIC ≤ 2 μg/mL were categorized as susceptible, and those with an MIC ≥ 8 μg/mL were classified as resistant. To ensure the reliability and accuracy of testing, *Escherichia coli* ATCC 25922 and *Pseudomonas aeruginosa* ATCC 27853 were employed as quality control organisms. Following inoculation, plates were incubated at 35 °C for 16–18 h.

### 2.5 Statistical analysis

Statistical analyses were performed using SPSS 27.0. The data processing and statistical analysis were completed between March and May 2026. Between-group comparisons of continuous variables (length of hospital stay, total drug costs, and antimicrobial drug costs) were conducted using the Kruskal–Wallis H test, and categorical outcomes were compared using the chi-square test. Variables significant in univariate analysis were entered into multivariate regression models adjusting for confounders, including age, underlying comorbidities, severity of infection, ICU admission, invasive procedures, source control, antimicrobial therapy, carbapenemase type, and study year. Length of stay was analyzed using Cox regression, with the proportional hazards assumption verified by log-cumulative survival plots; drug costs were natural log-transformed and analyzed using linear regression, with normality and homoscedasticity assessed by residual plots; clinical outcomes were analyzed using binary logistic regression, with model fit evaluated by the Hosmer–Lemeshow test and multicollinearity by variance inflation factor (VIF < 5). For categorical variables with statistical significance in multivariate analyses, pairwise comparisons were performed using the Bonferroni correction, with adjusted *p* < 0.05 considered statistically significant. Sensitivity analyses were conducted by combining adjacent groups. Differences in CRKP distribution were compared using the chi-square test, with the significance level set at α = 0.05. Antimicrobial susceptibility data were analyzed using WHONET 5.6 software.

### 2.6 Ethics statement

Approval for this study was granted by the Research Ethics Committee of the Second Hospital at Shanxi Medical University (YX2026026). The research involved a retrospective examination, and gathering of medical records and patient data were conducted. As the study was conducted observationally and utilized anonymous records, it was determined that informed consent from individual patients was not necessary.

## 3 Results

### 3.1 Phenotypic confirmation and changing trends of Carbapenemases

A total of 324 patients with CRKP infections were enrolled. The enrollment flow diagram of the patients is shown in Figure 1. Detailed information on the isolation rate of tested strains over the 10 year period is shown in Supplementary Table S1. Carbapenemase phenotyping was performed using the modified carbapenem inactivation method (mCIM) and the EDTA-modified carbapenem inactivation method (eCIM). We detected carbapenemase-encoding genes were detected by polymerase chain reaction (PCR). The testing results indicated that 108 isolates (33.33%) were positive for NDM, while 202 isolates (62.35%) were positive for KPC. Ten isolates did not produce a detectable carbapenemase. Notably, four CRKP isolates were found to co-produce both KPC and NDM carbapenemases. Between 2015 and 2020, NDM was the predominant carbapenemase among CRKP strains, accounting for an average of 70.24% annually. This trend shifted rapidly after 2021. From 2021 to 2024, KPC became the dominant carbapenemase, with its average annual proportion rising to 84.70%. We used a multiple regression discontinuity model to validate this transition. After adjusting for covariates including ward distribution, specimen type, and length of hospital stay, we found that in 2021, the proportion of KPC-producing CRKP strains exhibited a stepwise increase of 41.53% (95% CI: −7.16 to 90.18%, *p* = 0.077), while the proportion of NDM-producing strains showed a stepwise decrease of 46.06% (95% CI: −87.73% to −4.39%, *p* = 0.037). The adjusted regression discontinuity analysis confirmed that the shift in the KPC/NDM subtype composition in 2021 was statistically significant after controlling for the above confounding factors. Detailed data are presented in Table 1.

**Figure 1:**
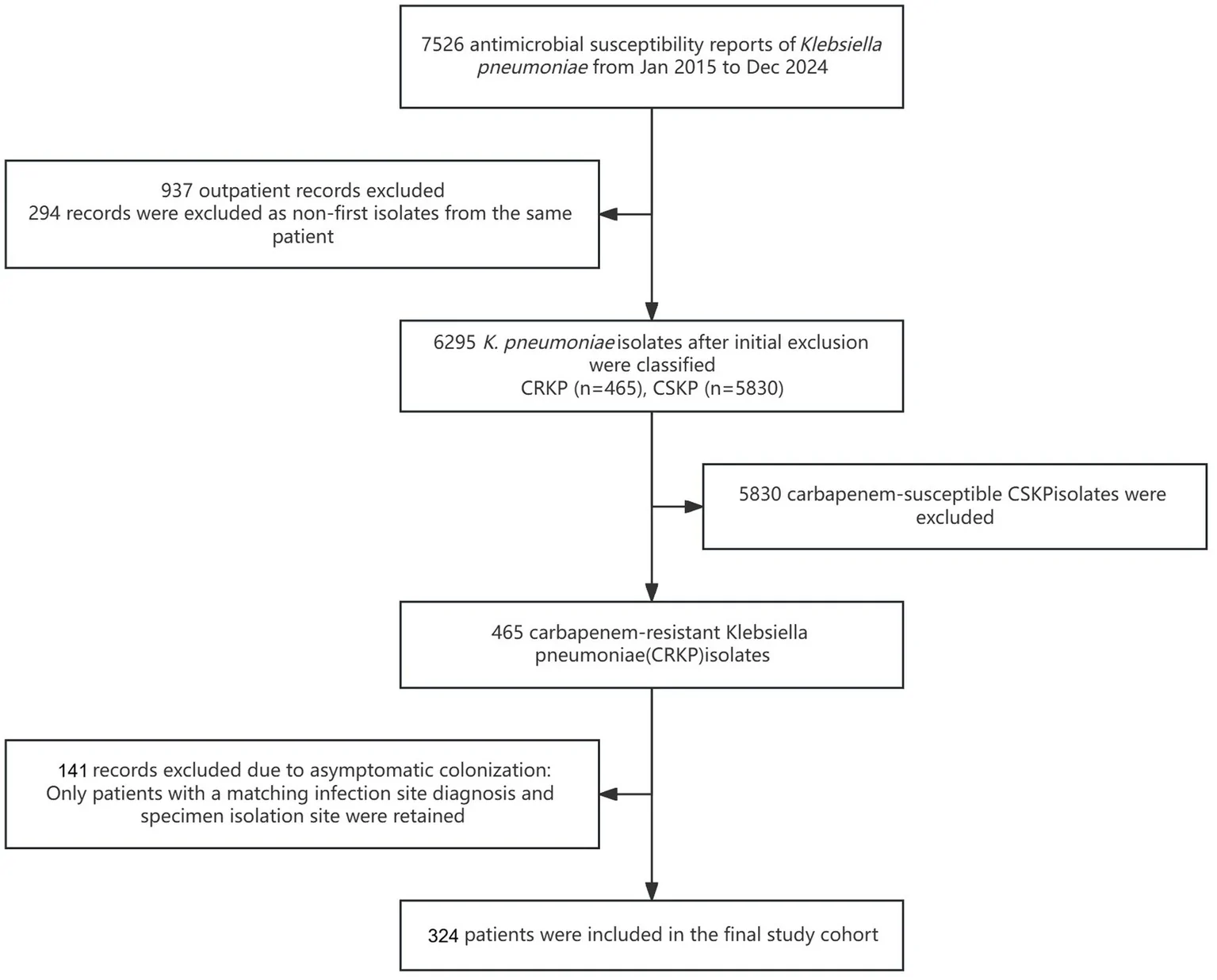
Flow diagram of carbapenem-resistant *Klebsiella pneumoniae* isolate selection, classification, and study cohort construction. CSKP: Carbapenem-Susceptible *Klebsiella pneumoniae*; CRKP: carbapenem-resistant *Klebsiella pneumoniae*.

**Table 1:** Multiple regression discontinuity analysis of changes in CRKP carbapenemase subtype proportions at the 2021 breakpoint.

Outcome mesure	Step change (%)	95% CI (%)	*p*	Adjusted covariates
Proportion of KPC-producing CRKP isolates	+41.53	−7.16 - 90.18%	0.077	Ward distribution, specimen type, length of hospital stay
Proportion of NDM-producing CRKP isolates	−46.06	−87.73% - -4.39%	0.037	

### 3.2 Antibiotic susceptibility testing in CRKP isolates

All 324 CRKP isolates were resistant to Meropenem and Imipenem (324/324, 100.00%). Among them, 184 were resistant to Cefoperazone-Sulbactam (184/324, 56.79%), 85 to Aztreonam (85/324, 26.23%), 134 to Amikacin (134/324, 41.35%), 29 to Tigecycline (29/324, 8.95%), and 4 to Colistin (4/324, 1.23%). Detailed data are presented in Table 2. The PCR results of the 324 CRKP strains are shown in Supplementary Table S2.

**Table 2:** *In vitro* susceptibility of CRKP isolates.

Antibiotic name	*Klebsiella pneumoniae* (*n* = 324)				
	R%	I%	S%	MIC_50_(mg/L)	MIC_90_(mg/L)
Meropenem	100 (324)	0	0	≥16	≥16
Imipenem	100 (324)	0	0	≥16	≥16
Cefoperazone-Sulbactam	56.79 (184)	0.31 (1)	42.90 (139)	≥16	≥64
Aztreonam	26.23 (85)	0.31 (1)	73.46 (238)	≥64	≥64
Amikacin	41.35 (134)	0.93 (3)	57.72 (187)	≥64	≥64
Tigecycline	8.95 (29)	2.78 (9)	88.27 (286)	2	8
Colistin	1.23 (4)	0	98.76 (320)	0.25	0.5

MIC_50/90_, 50%/90% minimum inhibitory concentration (mg/L); R%, percentage of resistant isolates; S%, percentage of susceptible isolates; I%, percentage of intermediary isolates.

### 3.3 Distribution of CRKP patients across four categories

Patient distribution was analyzed based on four criteria: the specimen type from which CRKP was isolated, disease severity, infection site, and the admitting hospital department. The annual distribution of severe CRKP cases is summarized in Figure 1. Sepsis and severe pneumonia accounted for 18.21% (59/324) and 19.14% (62/324) of all patients, respectively. A significant increasing trend was observed in the proportion of sepsis among CRKP patients over the study period (*χ*^2^_trend_ = 4.794, *p* = 0.029). As shown in Figure 2, sputum yielded the highest number of CRKP isolates, accounting for 63.58% (206/324), followed by urine and blood at 12.04% (39/324) and 6.48% (21/324), respectively. No significant temporal changes were observed in the detection rates of CRKP across the seven specimen categories. The annual distribution of CRKP infection types is presented in Figure 3. Pulmonary infection was the most prevalent type, accounting for 63.58% (206/324) of all CRKP patients, followed by bloodstream infection at 12.04% (39/324). The proportion of other infection types (each with fewer than 5 cases) demonstrated an increasing annual trend (*χ*^2^_trend_ = 19.617, *p* < 0.001). Figure 4 presents the annual departmental distribution of CRKP patients from 2015 to 2024. The distribution of CRKP patients across hospital departments remained relatively balanced, with the intensive care unit (ICU) accounting for the highest proportion at 24.07% (78/324), indicating generally severe clinical presentations among CRKP-infected individuals. Additionally, the proportions of CRKP patients admitted to the neurology and nephrology departments demonstrated an increasing annual trend during the study period (*χ*^2^_trend1_ = 8.721, *p* < 0.001, *χ*^2^_trend2_ = 5.432, *p* = 0.013; Figure 5).

**Figure 2:**
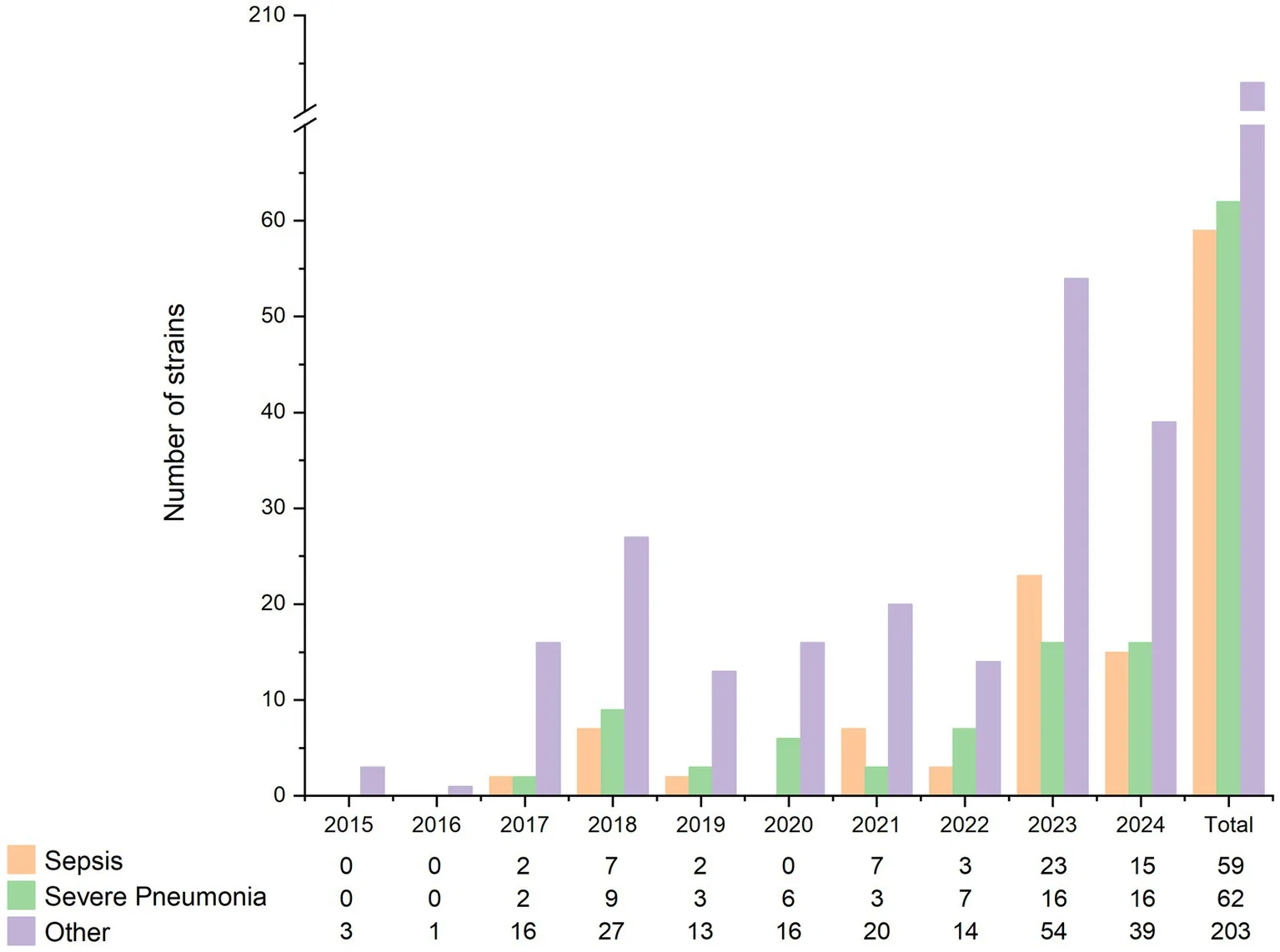
Severity distribution of associated with CRKP in a hospital (2015–2024).

**Figure 3:**
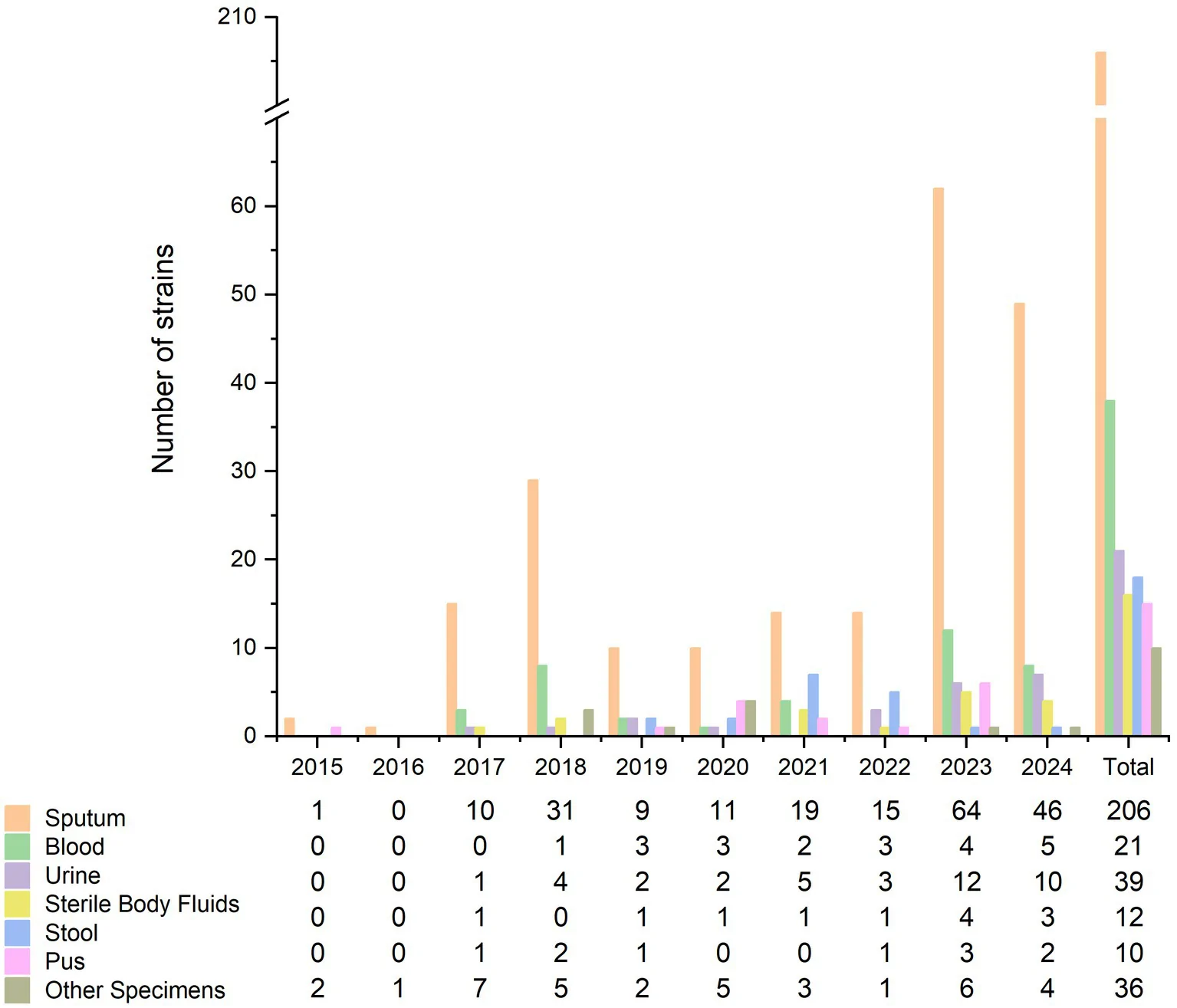
Specimen distribution of clinically isolated CRKP in a hospital (2015–2024).

**Figure 4:**
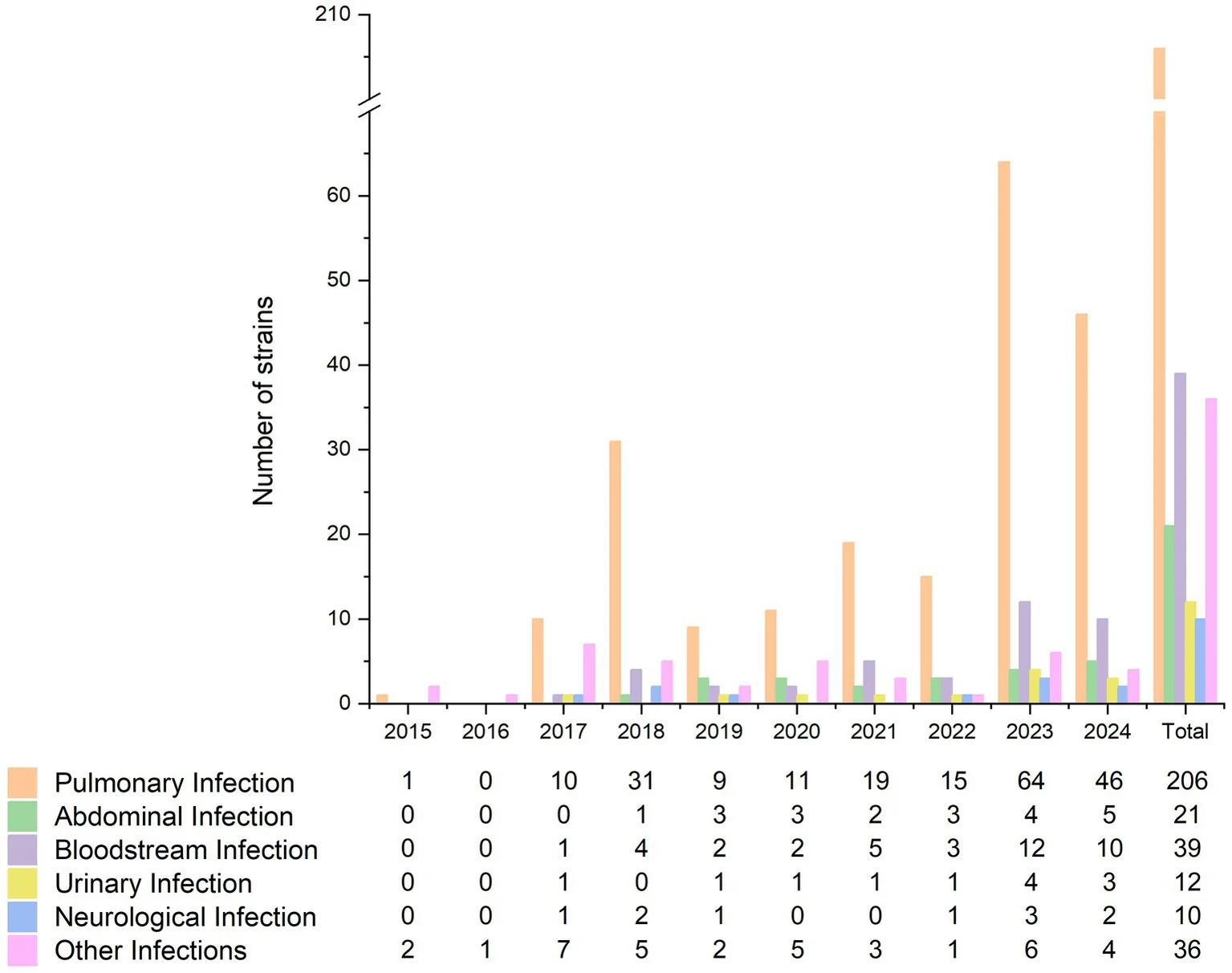
Disease distribution of clinically isolated CRKP in a hospital (2015–2024).

**Figure 5:**
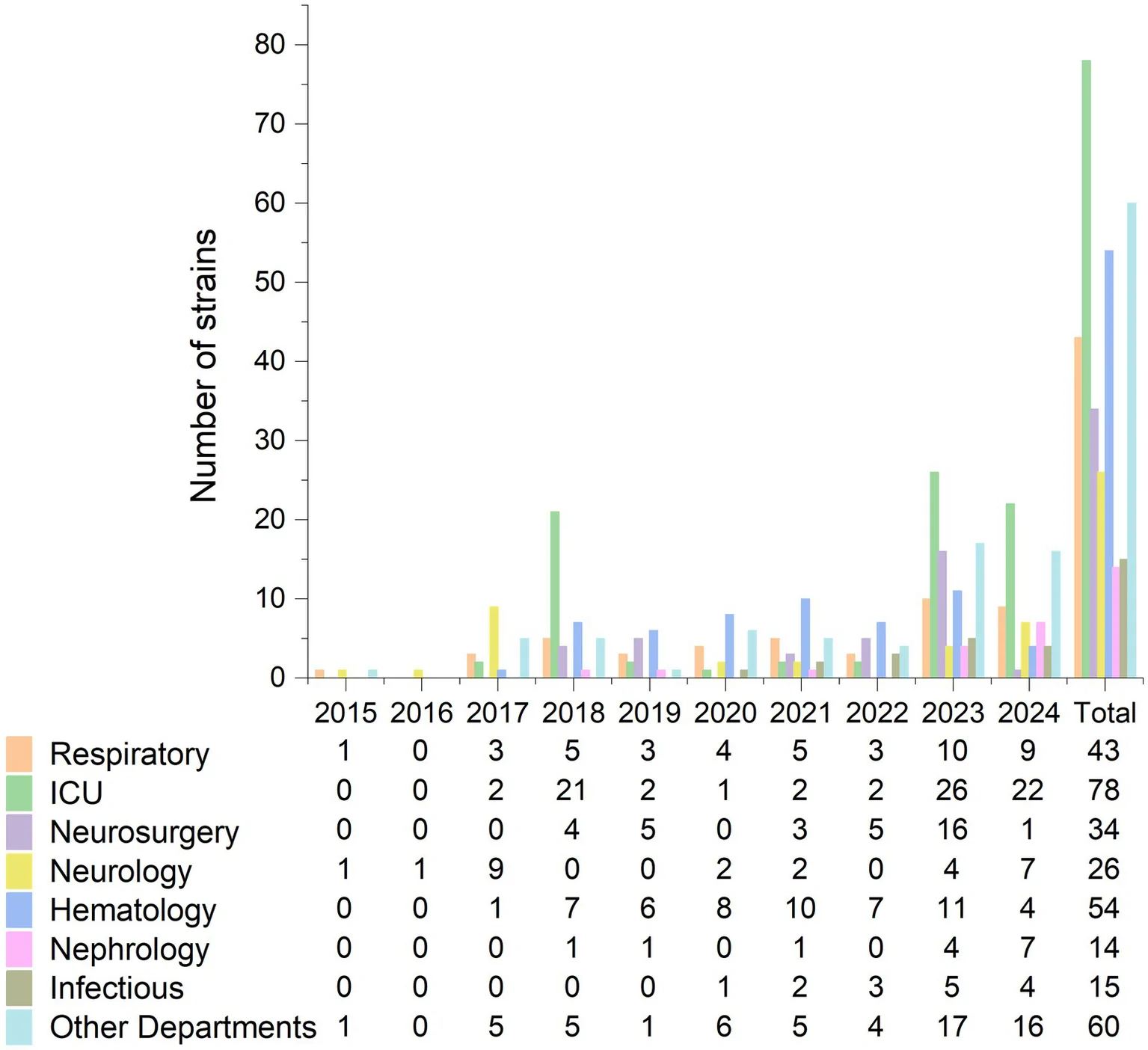
Departmental distribution of clinically isolated CRKP in a hospital (2015–2024).

Hospitalization expenses of all inpatients were denominated in Chinese Yuan (CNY), representing the actual expenses incurred during hospitalization, with the pricing base year being the actual charging standard of the discharge year. The median total drug cost was CNY 99,198.35 (IQR: CNY 45,770.65–197,347.13), and the median antimicrobial drug cost was CNY 6,932.51 (IQR: CNY 2,521.48–17,441.01). Due to the implementation of the zero-profit drug policy in 2017 and the centralized drug procurement policy in 2019, drug prices changed significantly over the 10-year period. Therefore, no cross-year inflation adjustment was applied to the costs in this study. Cost changes should be interpreted with caution.

### 3.4 Comparative analysis of CRKP patient distribution across groups

We conducted intergroup non-parametric comparisons of length of hospital stay, total drug cost, antimicrobial drug cost and treatment outcome in CRKP patients according to the above grouping, and the chi-square test was performed for treatment outcome. Besides, We conducted a sparsity subgroup sensitivity analysis by merging clinically similar subgroups. The re-analyzed results demonstrated that the direction of regression coefficients and significance levels were consistent between the original and merged datasets, indicating that the study results have satisfactory statistical robustness. The statistical results summarized in Table 2. Unadjusted univariate analysis showed that significant differences in hospitalization duration and antimicrobial drug costs were observed within the specimen-based grouping (*χ*^2^ = 23.589, *p* < 0.001; *χ*^2^ = 14.811, *p* = 0.022). For the severity-based grouping, statistically significant variations were identified in total medication costs, antimicrobial drug costs, and clinical outcomes (*χ*^2^ = 9.571, *p* = 0.023; *χ*^2^ = 20.711, *p* < 0.001; *χ*^2^ = 14.729, *p* < 0.001). Within the department-based grouping, only total medication costs showed a statistically significant difference (*χ*^2^ = 45.711, *p* < 0.001). In the disease-type grouping, significant differences were found in hospitalization duration, total medication costs, and antimicrobial drug costs (*χ*^2^ = 20.899, *p* < 0.001; *χ*^2^ = 21.224, *p* < 0.001; *χ*^2^ = 22.916, *p* < 0.001).

### 3.5 Multivariable regression analysis on CRKP patient distribution

Following the preliminary between-group comparisons, we first performed multivariate regression analysis, with age, underlying diseases, infection severity, ICU admission, invasive procedures, infection source control, antimicrobial therapy, carbapenemase type, and study year included as potential confounding factors. For length of hospital stay, a multivariate Cox regression model was used. The time origin is defined as the admission day of the patient’s current hospitalization, and the endpoint is defined as the all-cause patient discharge event occurring before the end of the observation period. For total drug cost and antimicrobial drug cost, which showed a positively skewed distribution, natural logarithmic transformation was performed followed by a multivariate linear regression model. For patient treatment outcome, a multivariate binary Logistic regression model was used. In terms of length of hospital stay, we found that after adjusting for confounding factors, disease classification had no significant independent effect on length of hospital stay (*p* = 0.145 > 0.05). Specimen classification showed a marginally significant trend with length of hospital stay (*p* = 0.077). Covariate analysis showed that invasive procedures were an independent influencing factor for length of hospital stay (HR = 1.74, 95% CI: 1.22–2.48, *p* = 0.002), and were associated with prolonged hospital stay. The above Cox regression models all satisfied the proportional hazards assumption. In terms of total drug cost, covariate analysis showed that symptom classification, disease classification, and department classification were independent influencing factors for total drug cost (P_symptom_ = 0.036, partial *η*^2^ = 0.017; P_disease_ < 0.001, partial η^2^ = 0.015; P_department_ < 0.001, partial *η*^2^ = 0.084). Meanwhile, in both symptom and disease classification models, ICU admission was also an independent influencing factor for total drug cost (*p* = 0.030, partial *η*^2^ = 0.015). The total drug cost of ICU-admitted patients was 1.37 times that of non-ICU-admitted patients (95% CI: 1.03–1.83). In terms of antimicrobial drug cost, symptom classification and disease classification were independent influencing factors for antimicrobial drug cost (P_symptom_ = 0.002, partial *η*^2^ = 0.039; P_disease_ < 0.001, partial *η*^2^ = 0.083). Specimen classification showed no significant independent effect on antimicrobial drug cost after adjustment for confounding factors (*p* = 0.082 > 0.05). The regression analyses of total drug cost and antimicrobial drug cost across all groups both satisfied normality and homogeneity of variance after logarithmic transformation. In terms of treatment outcome, binary Logistic regression analysis adjusting for confounding factors showed that only symptom classification was an independent influencing factor for treatment outcome (*p* = 0.005). The Hosmer-Lemeshow test yielded a *p* value of 0.12 (> 0.05), indicating good model fit. The VIF values of the above regression models were all less than 5, suggesting no severe multicollinearity (Table 3).

**Table 3:** Statistical results of between-group differences for the four CRKP classifications.

Between-grou*p* variance	Length of hospital stay		Total medication costs		Antimicrobial drug costs		Clinical outcomes	
	*χ* ^2^	*p*	*χ* ^2^	*p*	*χ* ^2^	*p*	*χ* ^2^	*p*
Severity distribution	5.442	0.142	9.571	0.023	20.711	<0,001	14.729	<0.001
Disease distribution	20.899	<0.001	21.224	<0.001	22.916	<0.001	2.834	0.726
Departmental distribution	11.84	0.106	45.711	<0.001	12.513	0.085	8.315	0.225
Specimen distribution	23.586	<0.001	9.276	0.159	14.811	0.022	5.437	0.489

Intergroup non-parametric tests are used for length of hospital stay and total drug cost and antimicrobial drug cost. The chi-square test is used for treatment outcome.

### 3.6 Within-group comparisons for CRKP patient distribution

We further conducted pairwise comparisons within the classification groups showing statistically significant differences using Mann–Whitney U test with Bonferroni-corrected multiple comparisons, aiming to identify which specific pairs of groups had significant differences in the data. Three pairwise comparisons were performed for symptom classification, 15 for disease classification, 28 for specimen classification, and 21 for department classification. The statistical results are presented in Tables 4–7. In terms of length of hospital stay, a significant difference in hospital stay duration was found between the sterile body fluid group and the sputum group under specimen classification (*p* = 0.010). The all-cause discharge risk of the sputum infection group is 2.44 times that of the sterile body fluid infection group, with a shorter overall length of hospital stay. (95% CI: 1.23–4.80). Since bacteria are usually detected in sterile body fluids only in severe infections, the longer hospital stay in the sterile body fluid group is consistent with this phenomenon. In terms of total drug cost, a significant difference was found in total drug cost between the sepsis group and the other groups under symptom classification (*p* = 0.014). The total drug cost in the sepsis group was 1.53 times that in the other groups (95% CI: 1.09–2.16). For disease classification, a significant difference was found in total drug cost between the pulmonary infection group and the urinary system infection group (*p* = 0.006). The total drug cost in the pulmonary infection group was 2.39 times that in the urinary system infection group (95% CI: 1.16–4.92). Under department classification, the total drug cost of neurosurgery patients was significantly different from that of the respiratory department, hematology department, and other departments (P1 < 0.001; P2 = 0.031; P3 = 0.023). The total drug cost of neurosurgery patients was 2.76 times (95% CI: 1.27–6.01), 2.09 times (95% CI: 1.03–4.26), and 2.05 times (95% CI: 1.05–4.01) that of the three latter departments, respectively. In terms of antimicrobial drug cost, under the symptom classification dimension, there was a significant difference in antimicrobial drug cost between the sepsis group and the other groups; the total drug cost of the sepsis group was 2.82 times that of the other groups (95% CI: 1.29–6.14, *p* = 0.004). Under the disease classification dimension, the antimicrobial drug cost of the pulmonary infection group was 5.64 times that of the urinary system infection group (95% CI: 1.36–23.34, *p* = 0.005) and 7.91 times that of the other infection group (95% CI: 1.93–32.40, *p* < 0.001), respectively. The antimicrobial drug cost of the abdominal infection group was 7.39 times that of the urinary system infection group (95% CI: 1.10–49.55, *p* = 0.031) and 10.36 times that of the other infection group (95% CI: 1.71–62.93, *p* = 0.002). The antimicrobial drug cost of the nervous system infection group also significantly differed from the total drug cost of the other infection group (*p* = 0.034), with the total drug cost of the nervous system infection group being 13.82 times that of the other group (95% CI: 1.11–172.27). In terms of treatment outcome, only the risk of adverse outcome in the other group was 32.3% of that in the sepsis group (95% CI: 15.8–65.8%, *p* = 0.002).

**Table 4:** Analysis of length of hospital stay in patients with clinically isolated CRKP strains in a hospital (2015–2024).

Comparative groups	HR	95% CI for HR	*p*	df
Disease types	/	/	0.145	5
Specimen types	/	/	0.077	6
Invasive procedure	1.74	1.22–2.48	0.002	/
Sputum-sterile body fluids	2.44	1.23–4.80	0.010	/

**Table 5:** Analysis of total medication costs in patients with clinically isolated CRKP strains in a hospital (2015–2024).

Comparative groups	RR	95% CI for RR	*p*	df
Severity types	/	/	0.036	2
ICU admission	1.36	1.03–1.79	0.030	2
Sepsis group-other group	1.54	1.09–2.16	0.014	/
Disease types	/	/	<0.001	5
ICU admission	1.37	1.03–1.83	0.030	5
Pulmonary infection-urinary tract infection	2.39	1.16–4.92	0.006	/
Departmental types	/	/	<0.001	7
Neurosurgery-respiratory medicine	2.76	1.27–6.01	<0.001	/
Neurosurgery-hematology	2.09	1.03–4.26	0.031	/
Neurosurgery- other departments	2.05	1.05–4.01	0.023	/

Sepsis Group: includes sepsis, bacteremia, septicemia, septic shock, and septic toxic shock. Other group: cases with milder infection. Other Departments: departments with fewer than 5 included patients.

**Table 6:** Analysis of antimicrobial drug costs in patients with clinically isolated CRKP strains in a hospital (2015–2024).

Comparative groups	RR	95% CI for RR	95% CI for RR	df
Severity types	/	/	0.002	2
Sepsis group- other group	2.82	1.29–6.14	0.004	/
Disease types	/	/	<0.001	5
Pulmonary infection-urinary tract infection	5.64	1.36–23.34	0.005	/
Pulmonary infection-other infections	7.91	1.93 ~ 32.40	<0.001	/
Abdominal infection-urinary tract infection	7.39	1.10–49.55	0.031	/
Abdominal infection-other infections	10.36	1.71 ~ 62.93	0.002	/
Neurological infection-other infections	13.82	1.11 ~ 172.27	0.034	/
Specimen types	/	/	0.082	6

Sepsis Group: includes sepsis, bacteremia, septicemia, septic shock, and septic toxic shock. Other group: the cases with milder infection. Other infections: infection sites with fewer than 5 included cases.

**Table 7:** Analysis of clinical outcomes in patients with clinically isolated CRKP strains in a hospital (2015–2024).

Comparative groups	OR	95%CI for OR	*p*	df
Severity types	/	/	0.005	2
Other group-sepsis group	0.32	0.16–0.66	0.002	/

Sepsis Group: includes sepsis, bacteremia, septicemia, septic shock, and septic toxic shock. Other group: cases with milder infection.

## 4 Discussion

Due to its rapid spread, CRKP has been regarded as a major global public health issue (21). At the same time, the epidemic characteristics of CRKP vary in different countries, regions and hospitals around the world (22). In our hospital, the number of isolates of CRKP strains continues to rise, which brings greater challenges to clinical treatment.

This study shows that sputum is the main specimen type for CRKP strains (62.96%), followed by blood (11.43%) and urine (10%). Moreover, the detection proportion in specimens of sputum specimens has always been dominant. This result suggests that CRKP infections are still mainly respiratory tract infections, which is consistent with the results of previous research reports (23). Among the infection sites, pulmonary infections account for 62.96% of the total number of patients, and bloodstream infections account for 12.04%. Although infections in other sites only account for 8.02%, they show an increasing trend year by year, indicating that the infection sites of CRKP are gradually diversifying, and it is necessary to strengthen the monitoring of infections in non-traditional sites (24). In previous studies, it was found that patients being admitted to the ICU may be an independent risk factor for CRKP infections (25). In our hospital, the department with the highest the number of CRKP isolates is the ICU (24.07%), which may be related to the widespread use of mechanical ventilation in the ICU, the low immunity of patients, and the high intensity of antibiotic use (26).

In this study, the CRKP strains collected showed extensive drug resistance, which was consistent with the monitoring data from other regions in previous studies (27). Notably, the number of highly drug-resistant strains with MIC≥16 increased significantly after 2023, suggesting that the drug resistance of CRKP may further intensify (28).

In terms of the change of carbapenemases, we found that the year 2021 was a critical turning point. Before 2021, the dominant type of carbapenemase was the NDM type (with an average proportion of 70.24% from 2015 to 2020). After that, the KPC type replaced the NDM type and became the mainstream carbapenemase produced by CRKP (with an average proportion of 84.70% from 2021 to 2024). This change is consistent with the epidemic trends in other regions of China (28). This change may be related to the adjustment of clinical drug-use strategies and patient mobility (29). Without whole-genome molecular typing data, we cannot fully rule out the possibility of transient clonal fluctuation, however, after adjusting for hospital-wide patient population, screening intensity and antimicrobial selection pressure, the 2021 carbapenemase prevalence shift was likely a population-level epidemiological transition rather than a single-cluster outbreak event. The KPC-type carbapenemase can easily cooperate with other drug - resistance genes. After it becomes the dominant type, it may lead to a further expansion of the drug-resistance spectrum of CRKP, bringing greater challenges to clinical treatment (30). At the same time, it also indicates an need to update the current testing methods for carbapenemases.

Multivariate regression analysis of inter-group comparisons showed that specimen type and infection site had a significant influence with a marginally significant trend on the length of hospital stay; infection site, hospitalization department classification, and severity degree were independent influencing factors that had a significant effect on total drug cost; infection site, severity degree, and specimen classification were independent influencing factors that had a significant effect on antimicrobial drug cost, while severity degree was the only significant independent influencing factor for clinical outcome.

And the severity of the illness is the only significant factor affecting the clinical outcome (severity of the illness = 17.287, *p* < 0.001). Further intra-group comparisons revealed a significant difference in length of hospital stay between patients with positive sterile body fluid specimens and patients in the respiratory department, which may be attributed to the fact that sterile body fluid infections mostly indicate deep or disseminated infections and require a longer treatment cycle than common respiratory tract infections. Invasive procedures were also found to be an independent influencing factor for length of hospital stay, suggesting that we should pay attention to the possible risk of CRKP infection in patients undergoing invasive procedures. ICU admission was an independent influencing factor for the increase in total drug cost, which is consistent with the result of a higher number of CRKP infections in the ICU. Meanwhile, significant differences were found in total drug cost, antimicrobial drug cost, and treatment outcome between sepsis patients and patients with common infections, which is related to the critical condition, high treatment difficulty, and the need for combined use of multiple high-priced antimicrobial drugs in sepsis patients. Significant differences were observed in total drug cost and antimicrobial drug cost between patients with pulmonary infection and patients with urinary system infection, and in antimicrobial drug cost between patients with abdominal infection and patients with urinary system infection. This reflects differences in disease severity and treatment needs across different infection sites. We also found that patients with nervous system infection and neurosurgery patients had significant differences in both total drug cost and antimicrobial drug cost compared with multiple departments and infection sites, suggesting that attention should be paid to CRKP infection in patients with nervous system infection. The total drug cost and antimicrobial drug cost of sepsis patients were significantly higher than those of other patients, and their clinical outcomes were worse, which is related to the critical condition, high treatment difficulty, and the need for combined use of multiple high-priced antimicrobial drugs in sepsis patients; the total drug cost of patients with positive sterile body fluid specimens was significantly higher than that of patients with sputum specimens, since sterile body fluid infections mostly indicate deep or disseminated infections and require a longer treatment cycle and higher costs than common respiratory tract infections; the length of hospital stay in patients with gastrointestinal infection and bloodstream infection was significantly longer than that in patients with urinary system infection, and the total drug cost in patients with bloodstream infection and pulmonary infection was significantly higher than that in patients with urinary system infection, reflecting differences in disease severity and treatment needs across different infection sites. At the department level, the total drug cost of patients in the neurology department was significantly higher than that in the respiratory medicine department, the total drug cost of ICU patients was significantly higher than that in the infectious diseases department, and the total drug cost of patients in the intensive care surgery department and neurosurgery department was significantly higher than that in the hematology department, which is related to differences in the complexity of underlying diseases and treatment regimens of patients admitted to different departments.

This study also has explicit limitations. It is a single-center, retrospective, observational study. The sample source and sample size are limited, so the results lack strong external validity and representativeness. And because the zero-markup drug policy reform in 2017 and the centralized drug procurement policies implemented since 2019 have exerted a substantial impact on drug prices, we did not perform cross-year inflation adjustment for the cost data, which introduces a notable limitation to the statistical analyses related to total drug cost and antimicrobial costs. We did not cover non-enzymatic resistance mechanisms. So our analysis of resistance mechanisms is not fully complete. We plan to conduct an additional independent multi-center validation in the future, to further investigate the applicability of the present findings to clinical decision-making.

## 5 Conclusion

In summary, we collected 324 CRKP strains from a tertiary hospital in Shanxi Province between 2015 and 2024. A shift in carbapenemase types occurred in 2021, when KPC replaced NDM as the dominant type. Most infections were respiratory, and sputum samples accounted for 62.96%, though the infection sites were becoming more varied. ICU patients made up the largest group (24.07%), and the proportion of sepsis cases rose each year. Infection site, ward, severity, and specimen type all had clear effects on length of stay and medical costs. Severity was the key factor shaping clinical outcomes.

## Data Availability

The original contributions presented in the study are included in the article/Supplementary material, further inquiries can be directed to the corresponding author/s.

## Footnotes

1 http://www.carss.cn/Report/Details?aId=808, accessed on 7 January 2026.

2
https://blast.ncbi.nlm.nih.gov/Blast.cgi
