# Supplementary material for: Trend of carbapenemase changes and epidemiological distribution of carbapenem-resistant Klebsiella pneumoniae clinically isolated in a hospital: a single-center retrospective study from 2015 to 2024
Source: Front Public Health. 2026 Oct 21;14:1944879. doi: 10.3389/fpubh.2026.1944879 (PMC13731328; doi:10.3389/fpubh.2026.1944879)
Supplement: Supplementary file 1 [file Supplementary_file_1.DOCX]

**Supplementary Table S1.** Statistics of number of clinical specimens, number of K. pneumoniae isolates, number of carbapenem-resistant isolates, hospital admissions, patient-days and CRKP Isolation Rate in a Hospital, 2015–2024

| Year | 2015 | 2016 | 2017 | 2018 | 2019 | 2020 | 2021 | 2022 | 2023 | 2024 | Total |
| --- | --- | --- | --- | --- | --- | --- | --- | --- | --- | --- | --- |
| Clinical specimens number | 2526 | 3006 | 3563 | 3879 | 4201 | 5272 | 6150 | 5837 | 8418 | 8547 | 51399 |
| KP isolates number | 325 | 377 | 520 | 638 | 647 | 744 | 965 | 824 | 1232 | 1254 | 7526 |
| CRKP number | 3 | 1 | 20 | 44 | 18 | 22 | 30 | 24 | 92 | 70 | 324 |
| Hospital admissions | 50441 | 50438 | 52216 | 61212 | 70013 | 61810 | 74801 | 75193 | 92586 | 100704 | 689414 |
| Patient-days | 550461 | 543979 | 545543 | 613741 | 667116 | 638301 | 694813 | 727517 | 723324 | 786615 | 6491409 |
| Isolation rate(%) | 0.12% | 0.03% | 0.56% | 1.13% | 0.43% | 0.42% | 0.49% | 0.41% | 1.09% | 0.82% | 0.63% |
| 95%CI(%) | 0.04%-0.34% | 0.004%-0.17% | 0.36%- 0.86% | 0.84%-1.52% | 0.27%-0.68% | 0.28%-0.63% | 0.34%-0.70% | 0.28%-0.61% | 0.89%-1.34% | 0.65%-1.04% | 0.57%-0.70% |

KP, *Klebsiella pneumoniae.* CRKP, Carbapenem-Resistant *Klebsiella pneumoniae.* The linear trend test showed that there was no statistically significant linear trend in the isolation rate over the study years. (t= 1.797, P=0.11>0.05).

**Supplementary Table S2.** Distribution of carbapenemase genes among 324 CRKP isolates

| Number | KPC | NDM | IMP | VIM | OXA-48 |
| --- | --- | --- | --- | --- | --- |
| 1 | + | - | - | - | - |
| 2 | + | - | - | - | - |
| 3 | + | - | - | - | - |
| 4 | + | - | - | - | - |
| 5 | + | - | - | - | - |
| 6 | - | + | - | - | - |
| 7 | + | - | - | - | - |
| 8 | + | - | - | - | - |
| 9 | + | - | - | - | - |
| 10 | + | - | - | - | - |
| 11 | + | - | - | - | - |
| 12 | + | - | - | - | - |
| 13 | + | - | - | - | - |
| 14 | + | - | - | - | - |
| 15 | + | - | - | - | - |
| 16 | + | - | - | - | - |
| 17 | + | - | - | - | - |
| 18 | + | - | - | - | - |
| 19 | + | - | - | - | - |
| 20 | + | - | - | - | - |
| 21 | + | - | - | - | - |
| 22 | + | - | - | - | - |
| 23 | + | - | - | - | - |
| 24 | + | - | - | - | - |
| 25 | - | + | - | - | - |
| 26 | + | - | - | - | - |
| 27 | + | - | - | - | - |
| 28 | + | - | - | - | - |
| 29 | + | - | - | - | - |
| 30 | + | - | - | - | - |
| 31 | + | - | - | - | - |
| 32 | + | - | - | - | - |
| 33 | + | - | - | - | - |
| 34 | + | - | - | - | - |
| 35 | + | - | - | - | - |
| 36 | - | + | - | - | - |
| 37 | + | - | - | - | - |
| 38 | + | - | - | - | - |
| 39 | + | - | - | - | - |
| 40 | + | - | - | - | - |
| 41 | + | - | - | - | - |
| 42 | + | - | - | - | - |
| 43 | - | + | - | - | - |
| 44 | + | - | - | - | - |
| 45 | + | - | - | - | - |
| 46 | + | - | - | - | - |
| 47 | + | - | - | - | - |
| 48 | + | - | - | - | - |
| 49 | - | + | - | - | - |
| 50 | + | - | - | + | - |
| 51 | + | - | - | - | - |
| 52 | + | - | - | - | - |
| 53 | + | - | - | - | - |
| 54 | - | + | - | - | - |
| 55 | + | - | - | - | - |
| 56 | + | + | - | - | - |
| 57 | + | - | - | - | - |
| 58 | + | - | - | - | - |
| 59 | + | - | - | - | - |
| 60 | + | - | + | - | - |
| 61 | - | + | - | - | - |
| 62 | + | - | - | - | - |
| 63 | + | - | - | - | - |
| 64 | + | - | - | - | - |
| 65 | + | - | - | - | - |
| 66 | + | - | - | - | - |
| 67 | + | - | - | - | - |
| 68 | + | - | - | - | - |
| 69 | + | - | - | - | - |
| 70 | + | - | - | - | - |
| 71 | + | - | - | - | - |
| 72 | + | - | - | - | - |
| 73 | + | - | - | - | - |
| 74 | + | - | - | - | - |
| 75 | - | + | - | - | - |
| 76 | + | - | - | - | - |
| 77 | + | - | - | - | - |
| 78 | + | - | - | - | - |
| 79 | + | - | - | - | - |
| 80 | + | - | - | - | - |
| 81 | + | - | - | - | - |
| 82 | + | - | - | - | - |
| 83 | + | - | - | - | - |
| 84 | + | - | - | - | - |
| 85 | + | - | - | - | - |
| 86 | + | - | - | - | - |
| 87 | + | - | - | - | - |
| 88 | + | - | - | - | - |
| 89 | + | - | - | - | - |
| 90 | + | - | - | - | - |
| 91 | + | - | - | - | - |
| 92 | + | - | - | - | - |
| 93 | + | - | - | - | - |
| 94 | + | - | - | - | - |
| 95 | + | - | - | - | - |
| 96 | + | - | - | - | - |
| 97 | + | - | - | - | - |
| 98 | + | - | - | - | - |
| 99 | + | - | - | - | - |
| 100 | + | - | - | - | - |
| 101 | + | - | - | - | - |
| 102 | + | - | - | - | - |
| 103 | + | - | - | - | - |
| 104 | + | - | - | - | - |
| 105 | + | - | - | - | - |
| 106 | + | - | - | - | - |
| 107 | + | - | - | - | - |
| 108 | + | - | - | - | - |
| 109 | + | - | - | - | - |
| 110 | + | - | - | - | - |
| 111 | - | + | - | - | - |
| 112 | + | - | - | - | - |
| 113 | + | - | - | - | - |
| 114 | + | - | - | - | - |
| 115 | + | - | - | - | - |
| 116 | + | - | - | - | - |
| 117 | + | - | - | - | - |
| 118 | + | - | - | - | - |
| 119 | - | + | - | - | - |
| 120 | + | - | - | - | - |
| 121 | + | - | - | - | - |
| 122 | + | - | - | - | - |
| 123 | + | - | - | - | - |
| 124 | + | - | - | - | - |
| 125 | + | - | - | - | - |
| 126 | - | + | - | - | - |
| 127 | + | - | - | - | - |
| 128 | - | + | - | - | - |
| 129 | - | + | - | - | - |
| 130 | + | - | - | - | - |
| 131 | + | - | - | - | - |
| 132 | + | - | - | - | - |
| 133 | - | + | - | - | - |
| 134 | + | - | - | - | - |
| 135 | + | - | - | - | - |
| 136 | - | + | - | - | - |
| 137 | - | + | - | - | - |
| 138 | - | + | - | - | - |
| 139 | - | + | - | - | - |
| 140 | - | + | - | - | - |
| 141 | + | - | - | - | - |
| 142 | + | - | - | - | - |
| 143 | - | + | - | - | - |
| 144 | - | + | - | - | - |
| 145 | - | + | - | - | - |
| 146 | + | - | - | - | - |
| 147 | - | + | - | - | - |
| 148 | + | - | - | - | - |
| 149 | - | + | - | - | - |
| 150 | + | - | - | - | - |
| 151 | - | + | - | - | - |
| 152 | - | + | - | - | - |
| 153 | + | - | - | - | - |
| 154 | + | - | - | - | - |
| 155 | - | + | - | - | - |
| 156 | + | - | - | - | - |
| 157 | + | - | - | - | - |
| 158 | + | - | - | - | - |
| 159 | + | - | - | - | - |
| 160 | + | - | - | - | - |
| 161 | + | - | + | - | - |
| 162 | + | - | - | - | - |
| 163 | + | - | - | - | - |
| 164 | + | - | - | - | - |
| 165 | + | - | - | - | - |
| 166 | + | - | - | - | - |
| 167 | + | - | - | - | - |
| 168 | + | - | - | - | - |
| 169 | + | - | - | - | - |
| 170 | + | - | - | - | - |
| 171 | + | - | - | - | - |
| 172 | + | - | - | - | - |
| 173 | + | - | - | - | - |
| 174 | + | - | - | - | - |
| 175 | + | - | - | - | - |
| 176 | - | + | - | - | - |
| 177 | + | - | - | - | - |
| 178 | + | - | - | - | - |
| 179 | + | - | - | - | - |
| 180 | + | - | - | - | - |
| 181 | + | - | - | - | - |
| 182 | + | - | - | - | - |
| 183 | + | - | - | - | - |
| 184 | - | + | - | - | - |
| 185 | + | - | - | - | - |
| 186 | + | - | - | - | - |
| 187 | + | - | - | - | - |
| 188 | + | - | - | - | - |
| 189 | - | + | - | - | - |
| 190 | + | - | - | - | - |
| 191 | + | - | - | - | - |
| 192 | + | - | - | - | - |
| 193 | - | + | - | - | - |
| 194 | + | - | - | - | - |
| 195 | + | - | - | - | - |
| 196 | + | - | - | - | - |
| 197 | + | - | - | - | - |
| 198 | + | - | - | - | - |
| 199 | - | + | - | - | - |
| 200 | + | - | - | - | - |
| 201 | + | - | - | - | - |
| 202 | + | - | - | - | - |
| 203 | + | - | - | - | - |
| 204 | + | - | - | - | - |
| 205 | + | - | - | - | - |
| 206 | + | - | - | - | - |
| 207 | + | - | - | - | - |
| 208 | - | + | - | - | - |
| 209 | + | - | - | - | - |
| 210 | + | - | - | - | - |
| 211 | - | + | - | - | - |
| 212 | + | - | - | - | - |
| 213 | + | - | - | - | - |
| 214 | - | + | - | - | - |
| 215 | + | - | - | - | - |
| 216 | + | - | - | - | - |
| 217 | + | - | - | - | - |
| 218 | - | + | - | - | - |
| 219 | - | + | - | - | - |
| 220 | - | + | - | - | - |
| 221 | + | - | - | - | - |
| 222 | - | + | - | - | - |
| 223 | + | - | - | - | - |
| 224 | - | + | - | - | - |
| 225 | - | + | - | - | - |
| 226 | + | + | - | - | - |
| 227 | - | + | - | - | - |
| 228 | - | + | - | - | - |
| 229 | + | - | - | - | - |
| 230 | - | + | - | - | - |
| 231 | + | + | - | - | - |
| 232 | + | - | - | - | - |
| 233 | - | + | - | - | - |
| 234 | - | + | - | - | - |
| 235 | - | + | - | - | - |
| 236 | + | - | - | + | - |
| 237 | - | + | - | - | - |
| 238 | + | - | - | - | - |
| 239 | + | - | - | - | - |
| 240 | - | + | - | - | - |
| 241 | + | - | - | - | - |
| 242 | - | + | - | - | - |
| 243 | - | + | - | - | - |
| 244 | + | - | - | - | - |
| 245 | - | + | - | - | - |
| 246 | - | + | - | - | - |
| 247 | - | + | - | - | - |
| 248 | + | - | - | - | - |
| 249 | - | + | - | - | - |
| 250 | - | + | - | - | - |
| 251 | + | - | - | - | - |
| 252 | - | + | - | - | - |
| 253 | - | + | - | - | - |
| 254 | - | + | - | - | - |
| 255 | + | - | - | - | - |
| 256 | + | - | - | - | - |
| 257 | - | + | - | - | - |
| 258 | - | + | - | - | - |
| 259 | - | - | - | - | - |
| 260 | - | - | - | - | - |
| 261 | - | + | - | - | - |
| 262 | - | + | - | - | - |
| 263 | - | + | - | - | - |
| 264 | - | - | - | - | - |
| 265 | - | - | - | - | - |
| 266 | - | + | - | - | - |
| 267 | - | + | - | - | - |
| 268 | - | - | - | - | - |
| 269 | - | + | - | - | - |
| 270 | + | - | - | - | - |
| 271 | - | + | - | - | - |
| 272 | - | + | - | - | - |
| 273 | - | + | - | - | - |
| 274 | - | + | - | - | - |
| 275 | - | + | - | - | - |
| 276 | - | - | - | - | - |
| 277 | - | - | - | - | - |
| 278 | - | - | - | - | - |
| 279 | - | + | - | - | - |
| 280 | - | + | - | - | - |
| 281 | - | + | - | - | - |
| 282 | - | + | - | - | - |
| 283 | - | + | - | - | - |
| 284 | - | + | - | - | - |
| 285 | - | + | - | - | - |
| 286 | - | + | - | - | - |
| 287 | - | + | - | - | - |
| 288 | + | - | - | - | - |
| 289 | - | + | - | - | - |
| 290 | - | + | - | - | - |
| 291 | + | - | - | - | - |
| 292 | - | + | - | - | - |
| 293 | - | + | - | - | - |
| 294 | - | + | - | - | - |
| 295 | - | - | - | - | - |
| 296 | - | - | - | - | - |
| 297 | - | + | - | - | - |
| 298 | - | + | - | - | - |
| 299 | - | + | - | - | - |
| 300 | + | + | - | - | - |
| 301 | + | - | - | - | - |
| 302 | - | + | - | - | - |
| 303 | - | + | - | - | - |
| 304 | - | + | - | - | - |
| 305 | + | - | - | - | - |
| 306 | - | + | - | - | - |
| 307 | - | + | - | - | - |
| 308 | - | + | - | - | - |
| 309 | - | + | - | - | - |
| 310 | - | + | - | - | - |
| 311 | - | + | - | - | - |
| 312 | - | + | - | - | - |
| 313 | - | + | - | - | - |
| 314 | - | + | - | - | - |
| 315 | - | + | - | - | - |
| 316 | + | - | - | - | - |
| 317 | - | + | - | - | - |
| 318 | - | + | - | - | - |
| 319 | + | - | - | - | - |
| 320 | - | + | - | - | - |
| 321 | - | + | - | - | - |
| 322 | + | - | - | - | - |
| 323 | - | + | - | - | - |
| 324 | - | + | - | - | - |

+ indicates the presence of the gene, - indicates the absence of the gene
